# Identification of Causal Plasma Proteins in Hepatocellular Carcinoma via Two-Sample Mendelian Randomization and Integrative Transcriptomic‒Proteomic Analysis

**DOI:** 10.1158/2767-9764.CRC-24-0553

**Published:** 2025-03-12

**Authors:** Weihao Tang, Xiaoke Ma

**Affiliations:** 1 College of Liberal Arts and Sciences, University of Florida, Gainesville, Florida.; 2 School of Computer Science and Technology, Xidian University, Xi’an, China.

## Abstract

Hepatocellular carcinoma (HCC) poses a serious threat to human health. Identifying diagnostic and therapeutic targets from plasma proteins is essential for early HCC detection and treatment. Large-scale plasma proteomics data and Mendelian randomization (MR) offer opportunities for this type of research. Proteomic data from protein quantitative trait loci were obtained from the UK Biobank Pharma Proteomics Project. Two-sample MR was used to evaluate the causal association between plasma protein levels and HCC. Sensitivity, directionality, and colocalization analyses were conducted to validate the causal associations identified by MR. Robustness was further tested through colocalization analysis. The single-cell dataset GSE166635 was used to assess the expression of genes corresponding to proteins identified via MR. Potential drugs targeting these proteins were evaluated using the DSigDB/DrugBank databases and molecular docking. Seven proteins with potential causal associations with HCC were identified: ASS1, B2M, FUOM, GABARAPL1, ST8SIA1, STOML2, and KRT8. Among these genes, ASS1, KRT8, and STOML2 presented the strongest effects in the colocalization analyses. In a single-cell liver cancer dataset, ASS1, KRT8, and STOML2 were expressed mainly in hepatic progenitor cells and malignant cells, with KRT8 predominantly found in hepatic progenitor cells and playing a role in the oncogenesis of malignant liver cells. Potential drugs targeting ASS1 include arginine, aspartic acid, and citrulline; for KRT8, ambroxol, diltiazem, and amikacin; and for STOML2, chlortetracycline, chlorzoxazone, and dirithromycin. This study identified several novel causal plasma proteins, providing new insights into the early diagnosis and treatment of HCC.

**Significance:**

In this study, we identified several causal proteins in HCC using UK Biobank Pharma Proteomics Project proteomic data via two-sample MR. We performed colocalization and sensitivity analyses, utilized single-cell RNA sequencing data for validation, and discovered potential drugs through molecular docking.

## Introduction

Hepatocellular carcinoma (HCC) is a severe disease that poses a significant threat to human health, with a 5-year survival rate of less than 20% (1). Given the poor prognosis of HCC, early diagnosis and intervention are highly important. Therefore, the discovery of new biomarkers or therapeutic targets for early-stage HCC has become a key focus in medical research. Although α-fetoprotein (AFP) has long been recognized as a classic marker for hepatic cancer (2), the identification of additional potential markers or effective intervention targets is still elusive. Several attempts have been made in the scientific community to address this issue. Recent studies have reported new genes that may be associated with HCC. For example, FBXO28 has been found to suppress liver cancer invasion and metastasis (3), and N-cadherin may serve as a diagnostic marker to differentiate primary liver carcinomas from extrahepatic carcinomas (4). Moreover, the overexpression of A1CF has been implicated in promoting HCC (5), and RBM12 has been shown to regulate gene expression in HCC (6). Although these genes may play a role in the mechanisms underlying the development of liver cancer, their associations with HCC have not been confirmed in large-scale clinical studies, nor is there evidence of their detection in plasma. The early detection of new markers or targets in plasma would be particularly valuable for the diagnosis and treatment of liver cancer.

In recent years, emerging projects focusing on the human proteome have generated a wealth of data on protein quantitative trait loci (pQTL). These data can be utilized to study biomarkers, mechanisms, and pathogenesis of various diseases (7). For example, one team developed genetic scores composed of independently inherited variants in the genes ATP citrate lyase and HMGCR. These genetic scores were then used to examine their associations with plasma lipid levels, lipoprotein concentrations, and the risk of cardiovascular events and cancer. The team discovered that ATP citrate lyase plays a significant role in cardiovascular disease (8). Another team employed two-sample Mendelian randomization (MR) to investigate the associations between genetic instruments for cardiovascular disease-related proteins and the risk of gestational hypertension and preeclampsia. They identified HSP27 as strongly associated with hypertensive disorders of pregnancy (9).

In regard to HCC, research on the association between plasma proteins and HCC is still in its early stages. Previous studies have explored the association between the gut microbiome and primary liver cancer (10), as well as the associations between lipid indicators and liver cancer (11). However, from a proteomic perspective, studies of HCC are lacking as these individual research findings have not fully elucidated the complex role that plasma proteins play in HCC. Understanding the potential causality between proteins and disease could be a significant indicator for diagnosing HCC and implementing early intervention strategies. Therefore, it is crucial to utilize large-scale plasma protein data to screen and discover new pathogenic factors or diagnostic markers for HCC.

This study utilized large-scale plasma proteomics pQTL data and genome-wide association study (GWAS) data on liver cancer to identify potential circulating plasma proteins that could serve as diagnostic markers or therapeutic targets for HCC. We employed two-sample MR, colocalization, directionality analysis, sensitivity analysis, and external validation to ensure the robustness of the causal association between plasma proteins and HCC. Additionally, we examined a single-cell dataset of liver cancer to explore the expression patterns of proteins identified through MR in different types of cells (12). To further advance our understanding, we conducted drug screening and molecular docking to identify potential interventional drugs for the novel proteins identified. This study aims to provide novel data to support further investigations into early blood diagnosis, drug intervention targets, and the potential pathogenesis of HCC.

## Materials and Methods

### Overall design

**Figure 1:**
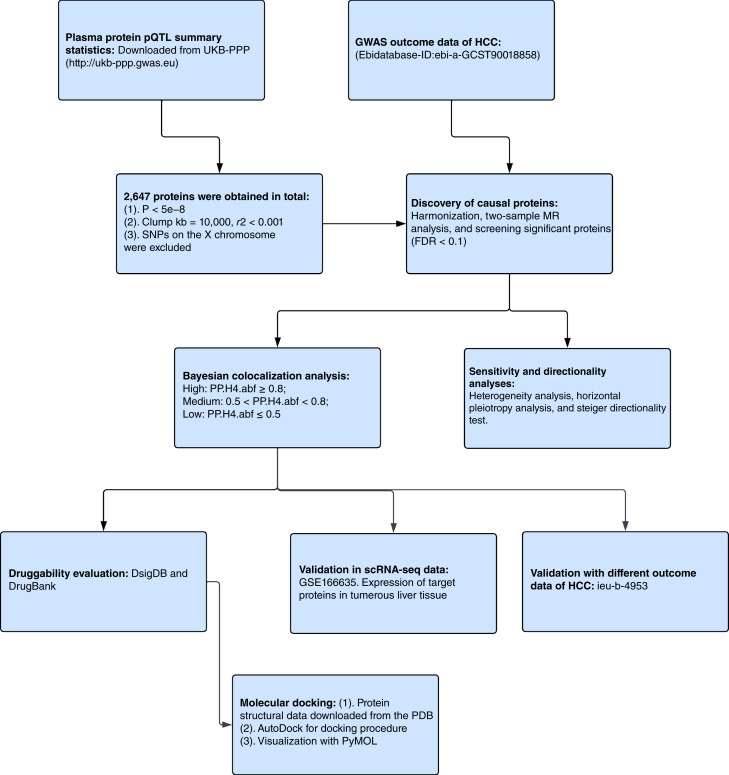
Flowchart of this study. PDB, Protein Data Bank; PP.H4.abf, H4 posterior probability.

The overall design of this study is depicted in Fig. 1. We utilized pQTL data from the UK Biobank Pharma Proteomics Project (UKB-PPP; RRID:SCR_026189; ref. 7) and analyzed their associations with HCC through two-sample MR. Colocalization analysis was employed to validate the associations between target proteins and HCC. Sensitivity analyses, including heterogeneity, pleiotropy, and random effect model (REM) analyses, were conducted to confirm the robustness of the causal associations of proteins. The Steiger directionality test (13) was used to ensure the direction of causality from proteins to the HCC. The results obtained from MR were then validated with external outcome data (ieu-b-4953). The single-cell RNA sequencing (scRNA-seq) dataset GSE166635 in Gene Expression Omnibus (RRID:SCR_005012; ref. 12) was used to analyze the expression of the corresponding genes of the screened proteins in the tumorous liver tissue. Finally, we evaluated potential interventional drugs that target the screened proteins. All data used in this study are publicly available, exempting it from the need for ethical review. Detailed download links of datasets, software, and other key resources are available in as well as characteristics of data used can be found in Supplementary Tables S1 and S2, respectively.

### Preparation of genetic instruments

The following criteria were applied to prepare the genetic instruments for MR: (i) pQTL summary-level data were downloaded from the UKB-PPP database (https://doi.org/10.7303/syn51364943); (ii) significant SNPs were selected (*P* < 5e−8); (iii) to address linkage disequilibrium, PLINK 1.9 (NITRC; RRID:SCR_001757) on the Linux system was employed, with parameters set to clump SNPs within 10,000 kb and *r*^2^ < 0.001; and (iv) SNPs on the X chromosome were excluded. In total, 2,647 pQTL datasets for proteins were obtained for MR.

### Preparation of outcome data for discovery

The outcome GWAS data were downloaded from the ieu open GWAS project *by* searching for HCC GWAS summary statistics. In this study, GCST90018858 (RRID:SCR_012745; ref. 14), a European population with 475,638 (ncase 379 and ncontrol 475,259) and 24,194,938 SNPs, was utilized as outcome GWAS data. The following columns were retained for further analysis: “variant_id,” “beta,” “standard_error,” “effect_allele,” “other_allele,” “effect_allele_frequency,” and “*P*_value.”

### Two-sample MR analysis

The R package TwoSampleMR 0.5.7 (MRCIEU; RRID:SCR_019010) was employed to conduct MR. The function “harmonize_data” was used to harmonize instruments and outcomes by joint SNPs between the discovery instrumental variables and GWAS outcome. Instruments with F statistics greater than 10 were retained for MR analysis. The associations between each protein and the outcome were calculated individually. The *P* value in the MR results was adjusted via the *FDR* method (15): *FDR* = *P* value × 2647. For proteins with only one SNP, the Wald ratio (WR) was used for the calculation, whereas the inverse variance weighted (IVW) method was utilized for MR when multiple SNPs were available. Significant MR results were identified on the basis of an *FDR* < 0.1.

### Validation of the causality based on external outcome data

The outcome data of ieu-b-4953 (RRID:SCR_012815; ref. 16), which represents a European population from a different cohort than the one used for discovery, were downloaded from the GWAS database (https://gwas.mrcieu.ac.uk/). The sample size for this dataset was 372,184 (168 cases and 372,016 controls), and it included 6,304,034 SNPs. These data were used as the external GWAS outcome for validation purposes. The same pQTLs of the target proteins that were used in the discovery phase were used as the exposure. To validate the causality between plasma proteins and HCC, a two-sample MR analysis was conducted using these GWAS outcome data. A *P* value of less than 0.05 was considered statistically significant.

### Sensitivity analyses and Steiger directionality test

The R package TwoSampleMR 0.5.7 (MRCIEU; RRID:SCR_019010) was used, specifically the function mr_heterogeneity, to assess heterogeneity for proteins with more than 3 SNPs. If heterogeneity was present (*q* < 0.05), MR analysis was recalculated via a REM to determine causality (*P* < 0.05 indicates causality). The function mr_pleiotropy was used to test for pleiotropy of the pQTLs (*P* > 0.05 indicates no pleiotropy for proteins with more than 3 SNPs). The Steiger directionality test was conducted via the function directionality_test (*P* < 0.05 indicates one-way directionality).

### Bayesian colocalization analysis

Various hypotheses have been incorporated into the Bayesian colocalization framework—H0: Phenotype 1 (GWAS) and phenotype 2 (pQTL) are not significantly associated with any SNP loci in a genomic region. H1/H2: Both phenotype 1 (GWAS) and phenotype 2 (pQTL) are significantly associated with SNP loci in a genomic region. H3: Both phenotype 1 (GWAS) and phenotype 2 (pQTL) are significantly associated with SNP loci in a genomic region but are driven by different causal variant loci. H4: Both phenotype 1 (GWAS) and phenotype 2 (pQTL) are significantly associated with SNP loci in a genomic region and are driven by the same causal variant locus.

The R package coloc 5.2.3 (RRID:SCR_026041; ref. 17) was used for colocalization analysis. Variance and minor allele frequency values were calculated for the GWAS outcome data. Variance, minor allele frequency, and z values were calculated for the pQTL data of the target proteins, and the leading SNP was identified on the basis of the *P* value. All SNPs within 1 Mb upstream and downstream of the leading SNP were selected for further analysis. Colocalization analysis was performed via the coloc.abf function, considering only the SNPs that were common to both the pQTL data and the GWAS outcome data. A colocalization association between a pQTL and GWAS was considered if the posterior probability (*PP.H4*.abf) was greater than 0.5. The posterior probability can be calculated for each of these hypotheses to summarize the genetic association data (18).

### Gene expression of screened proteins in scRNA-seq data

The dataset GSE166635 (12) was utilized to investigate the gene expression of proteins identified by MR in scRNA-seq data. For subsequent analysis, the Seurat 5.1.0 (RRID:SCR_016341; ref. 19) R package was employed. The standard workflow of normalization, finding variable features, and scaling the data were performed with default parameters. The cells were filtered on the basis of the following criteria: nFeature_RNA >500 and nCount_RNA >800. Principal component analysis was conducted using the first 10 principal components. Clusters were identified via the function FindClusters (resolution = 0.1) in SeuratV5. Uniform Manifold Approximation and Projection was then used for dimension reduction on the clusters (20). The marker genes were identified via the function FindAllMarkers, with a minimum percentage of 0.5 and a log-fold change threshold of 0.05. Cells were annotated on the basis of known marker genes specific to each cell lineage, including malignant cells (AFP and GPC3; ref. 21); tumor-associated macrophages (CD14, CD163, and C1QA); T cells (CD3E, CD3D, CD3G, and CCR1); hepatic progenitor cells (HPC; EPCAM, KRT19, and CD24); B cells (CD79A, CD37, and CD55); cancer-associated fibroblasts (COL1A2, BGN, and ACTA2); and tumor endothelial cells (PECAM1 and ENG; ref. 12). The Wilcoxon test (22) was performed between HPC-like cells and malignant cells for KRT8 on the basis of function stat_compare_means in R package ggpubr 0.6.0 (RRID:SCR_021139; ref. 23). Visualization was performed via functions in the R packages Seurat 5.1.0 (RRID:SCR_016341; ref. 19) and ggplot2 3.5.1 (Wickham H; RRID:SCR_014601).

### Identifying potential therapeutic drugs and molecular docking of MR proteins and drugs

Interventional drugs for the target proteins were evaluated via the DSigDB (RRID:SCR_026202; ref. 24) and DrugBank (RRID:SCR_002700; ref. 25) databases. These databases were searched using the respective names of the screened proteins identified via MR as keywords. The protein structural data were obtained from the Protein Data Bank (RRID:SCR_012820; ref. 26). AutoDockTools was used to set up the graphical user interface for AutoDock 4.2.6 (RRID:SCR_012746; ref. 27), which was used to prepare the docking procedure. A docking grid box with dimensions of 126 Å × 126 Å × 126 Å was defined, with a grid spacing of 0.375 Å. Docking calculations were performed via a genetic algorithm in AutoDock 4.2.6. During the docking simulation, each ligand was kept flexible, whereas the amino acid residues of the protein remained rigid. AutoDock 4.2.6 was used to perform the molecular docking procedure, and the binding energy was obtained as a reference indicator. The results were visualized via PyMOL 2.6.0 (Schrödinger LLC.; RRID:SCR_000305).

### IHC in the Human Protein Atlas

The Human Protein Atlas (HPA; RRID:SCR_006710; ref. 28) was utilized to evaluate the MR proteins in patients with HCC. Keywords of MR protein names were entered in the search box of the HPA website to obtain protein IHC images in normal and cancer tissues. The contrast between normal and liver cancer tissues were evaluated based on the staining extent and pattern.

### Data availability

All summary-level GWAS data and scRNA-seq data used in this study are publicly available, and the download links can be found in supplementary tables. The datasets generated during the current study are available from the corresponding author on reasonable request.

## Results

### Causal proteins of HCC discovered through two-sample MR

**Figure 2:**
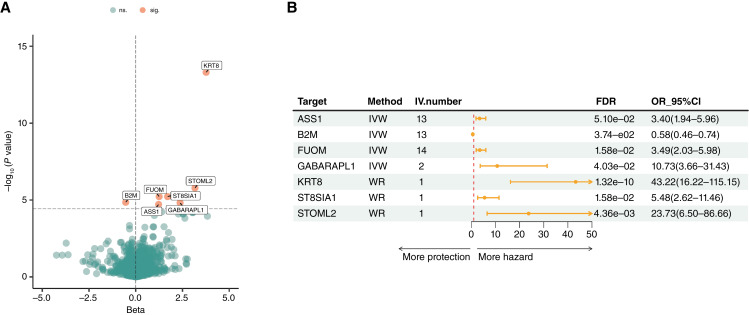
Results of MR analysis. **A,** Volcanic plot of MR results in the European population. Proteins with labels demonstrate significance (*P* value < 8.66e−6). ns., no statistical significance; sig., statistical significance. **B,** Forest plot of MR results of 7 proteome-wide identified proteins from European population GWAS. The error bar represents 95% CI of OR. IV.number, the number of SNP instrumental variables.

Seven significant plasma proteins were identified as causal proteins of HCC (Fig.2A), including ASS1 [Odds ratio (*OR*) = 3.40; 95% confidence interval (CI), 1.94–5.96; *FDR* = 5.10e−02], B2M (*OR* = 0.58; 95% CI, 0.46–0.74; *FDR* = 3.74e−02), FUOM (*OR* = 3.49; 95% CI, 2.03–5.98; *FDR* = 1.58e−02), GABARAPL1 (*OR* = 10.73; 95% CI, 3.66–31.43; *FDR* = 4.03e−02), ST8SIA1 (*OR* = 5.48; 95% CI, 2.62–11.46; *FDR* = 1.58e−02), STOML2 (*OR* = 23.73; 95% CI, 6.50–86.66; *FDR* = 4.36e−03), and KRT8 (*OR* = 43.22; 95% CI, 16.22–115.15; *FDR* = 1.32e−10). Among these proteins, ASS1, FUOM, GABARAPL1, ST8SIA1, STOML2, and KRT8 were found to be hazardous proteins in HCC (*OR* > 1), whereas B2M was identified as a protective protein (*OR* < 1; Fig.2B). Although KRT8 and STOML2 only have 1 SNP, the F statistics of SNP of these two proteins are 40.804 and 44.959, respectively. An F statistic value higher than 10 usually indicates the level of bias is small (29, 30). The results for all the MR proteins can be found in Supplementary Table S3.

### Sensitivity and directionality analyses of the identified proteins

The results of the sensitivity and directionality tests can be found in Table 1. All target proteins identified through MR passed the Steiger directionality test (Steiger *P* < 0.05; ref. 13), indicating that these proteins may be the causes of HCC. Among the 7 target proteins, ASS1, B2M, FUOM, and GABARAPL1 were further analyzed for their sensitivity. KRT8, ST8SIA1, and STOML2 were not able to undergo sensitivity analysis because of the existence of only 1 SNP. After heterogeneity analysis, the proteins ASS1 and FUOM showed heterogeneity (*Q* value < 0.05). Therefore, the REM was used to reconduct MR analysis on these proteins. Both proteins had a *P* value < 0.05 for the REM, indicating a significant association between blood plasma proteins and HCC. The proteins ASS1, B2M, and FUOM, with more than 3 SNPs, were tested for pleiotropy via the function mr_pleiotropy. The results revealed that none of these proteins had pleiotropy (*P* > 0.05).

**Table 1:** Results of MR directionality and sensitivity analyses

Target	Protein name	Method	Number of SNPs	Steiger *P* value	Heterogeneity *Q* value	REM *P* value	Pleiotropy *P* value
ASS1	Argininosuccinate synthase 1	IVW	13	5.05E−148	1.03E−04	1.93E−05	6.23E−01
B2M	β-2-Microglobulin	IVW	13	0.00E+00	7.88E−01	9.81E−08	4.67E−01
FUOM	Fucose mutarotase	IVW	14	4.53E−255	4.86E−04	5.98E−06	6.18E−01
GABARAPL1	GABA type A receptor–associated protein like 1	IVW	2	3.86E−13	3.74E−01	1.12E−06	N/A
KRT8	Keratin 8	WR	1	2.03E−05	N/A	N/A	N/A
ST8SIA1	ST8 α-N-acetyl-neuraminide α-2,8-sialyltransferase 1	WR	1	6.05E−10	N/A	N/A	N/A
STOML2	Stomatin-like 2	WR	1	1.36E−07	N/A	N/A	N/A

### Colocalization analysis

**Figure 3:**
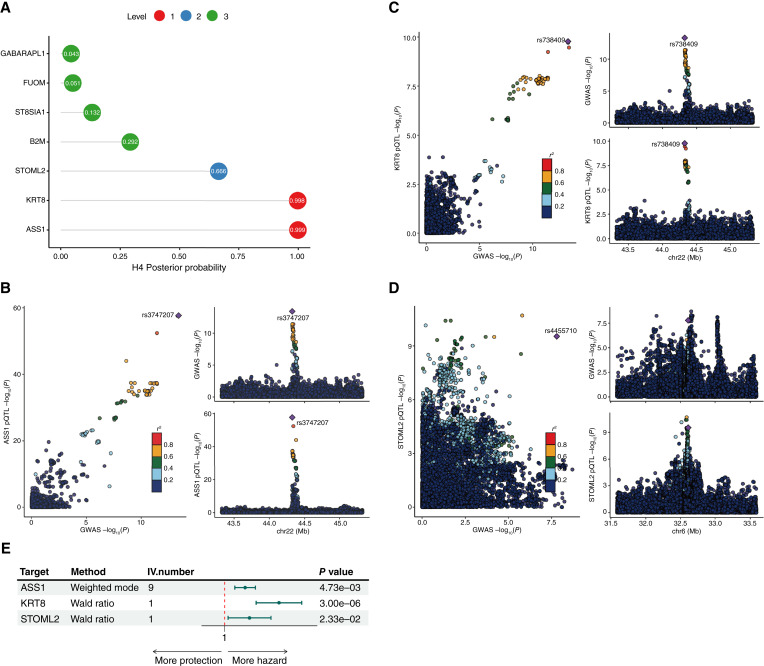
Results of colocalization and external validation. **A,** Colocalization results of 7 proteins identified by MR. Proteins were categorized based on H4 posterior probability in to 3 levels. (1) Level 1: ASS1 and KRT8 (*PP.H4* ≥ 0.8); (2) level 2: STOML2 (0.5 < *PP.H4* < 0.8); (3) level 3: B2M, FUOM, GABARAPL1, and ST8SIA1 (*PP.H4* ≤ 0). **B–D,** Locus comparison plots of joint SNPs in terms of the association between blood plasma proteins and HCC in the respective pQTL and GWAS data of ASS1, KRT8, and STOML2. The left displays the −log_10_*P* values for SNP associations with HCC outcomes along the *x*-axis and the −log_10_*P* values for associations with blood plasma proteins from the UKB-PPP along the *y*-axis. The right displays genomic positions on the *x*-axis, with −log_10_*P* values for the HCC outcome shown in the upper section and −log_10_*P* values for blood plasma proteins shown in the lower section on the *y*-axis. **E,** Forest plot of external outcome validation of 3 proteins passed through colocalization, ASS1, KRT8, and STOML2. The error bar represents 95% CI of OR. GWAS, Genome-wide association study.

Colocalization analysis was conducted on the seven proteins identified by MR in relation to HCC via the R package coloc (version 5.2.3; ref. 17). On the basis of the posterior probability of colocalization results, the proteins were categorized into three levels, indicating the degree to which exposure and outcomes share the same causal variance—Level 1: ASS1 and KRT8 were selected owing to their high posterior probability (*PP.H4* ≥ 0.8); level 2: STOML2 was classified on the basis of a medium posterior probability (0.5 < *PP.H4* < 0.8); level 3: GABARAPL1, FUOM, ST8SIA1, and B2M were grouped together owing to a low posterior probability (*PP.H4* ≤ 0.5; Fig.3A). Supplementary Table S4 contains all the colocalization results. We obtained evidence supporting the presence of shared causal variants between pQTLs and outcome data through colocalization analysis of genomic regions within 1 Mb of lead SNPs for proteins in tiers 1 and 2 (*PP.H4* > 0.5). The regional colocalization plots for these associations are presented in Fig. 3B–D. The lead SNPs rs3747207 (ASS1), rs738409 (KRT8), and rs4455710 (STOML2) were considered the most likely shared causal variants for the regions displaying colocalization evidence.

### Validation on external GWAS outcome data

For external validation, we utilized liver cancer GWAS outcome data from a different cohort (ieu-b-4953) to validate the three MR proteins ASS1, KRT8, and STOML2, which presented the highest posterior probability in the colocalization analysis (Fig. 3E). The validation results for all three proteins were consistent with those of the discovery cohort (ebi-a-GCST90018858), indicating that these proteins have a detrimental effect on the liver cancer outcome.

### Single-cell data exploration

**Figure 4:**
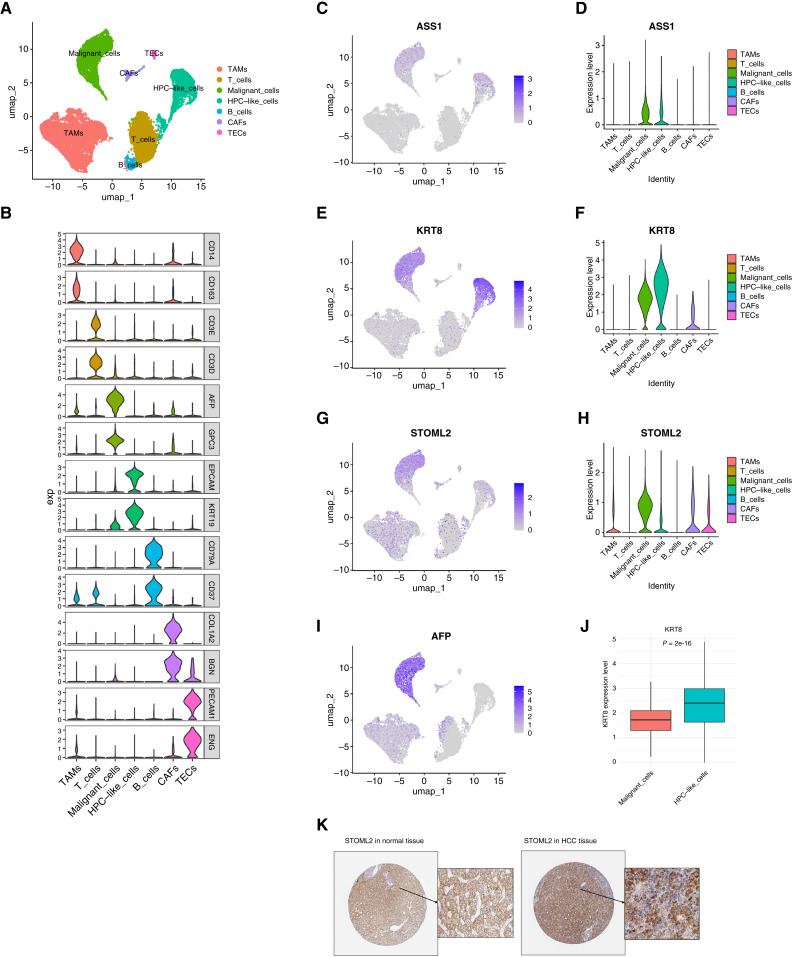
Expression of the target genes in single-cell clusters of liver cancer tissue. **A,** Uniform Manifold Approximation and Projection dimension reduction plot of single cells from liver tissue of patients with liver cancer. **B,** Marker genes for each cell cluster. **C,** Feature plot showing the distribution of ASS1 across cell clusters. **D,** Violin plot showing the distribution of ASS1 across cell clusters. **E,** Feature plot showing the distribution of KRT8 across cell clusters. **F,** Violin plot showing the distribution of KRT8 across cell clusters. **G,** Feature plot showing the distribution of STOML2 across cell clusters. **H,** Violin plot showing the distribution of STOML2 across cell clusters. **I,** Feature plot showing the distribution of the liver cancer marker AFP across cell clusters. **J,** Gene differential expression of KRT8 in HPCs and malignant cells. **K,** IHC images of STOML2 in normal liver tissues and HCC (Left, patient ID: 1720, male, normal; right, patient ID: 889, male, HCC). CAFs, cancer-associated fibroblasts; TAMs, tumor-associated macrophages; TECs, tumor endothelial cells.

The single-cell dataset was analyzed to show that the causal proteins in plasma might partly come from gene expression in liver cancer cells. In our analysis, a total of 7 cell types, including tumor-associated macrophages, T cells, malignant cells, HPCs, B cells, cancer-associated fibroblasts, and tumor endothelial cells, were clustered and annotated (Fig. 4A). Violin plots were used to display the marker genes utilized for cluster annotation (Fig. 4B). Additionally, feature plots and violin plots were generated for the tier 1 and tier 2 proteins ASS1, KRT8, and STOML2 and the liver cancer marker gene AFP (Fig. 4C–I). The results demonstrated that ASS1, KRT8, and STOML2 were expressed primarily in malignant cells and HPC-like cell clusters. STOML2 was partially expressed in HPC-like cells in addition to malignant cells, ASS1 was expressed in both HPC-like and malignant cells, and KRT8 was significantly expressed in the HPC-like cluster, independent of malignant cells. Furthermore, a difference was observed (*P* < 0.05) between HPC-like cells and malignant cells for KRT8 (Fig. 4J) via the Wilcoxon test (22). On the basis of the conclusions of the original paper by the authors on GSE166635 (12), which suggested that malignant cells originate from the HPC-like population, these three hazardous proteins, particularly KRT8, may play a significant role in the entire transformation process from HPCs to cancerous cells.

### Drugs for MR proteins and molecular docking of MR proteins and drugs

**Figure 5:**
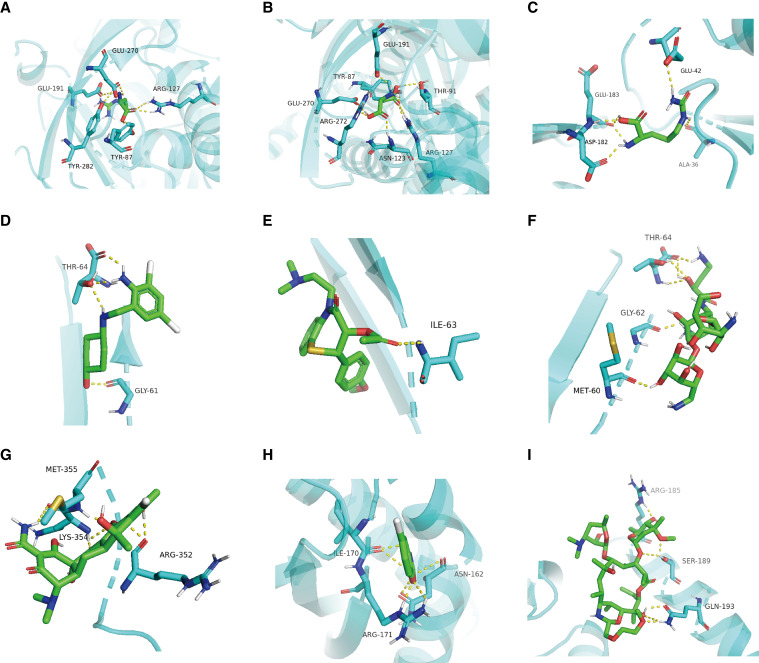
Molecular docking visualization between target proteins and drugs. **A**–**C,** Molecular docking plot of ASS1 with arginine, aspartic acid, and citrulline, respectively. **D–F,** Molecular docking plot of KRT8 with ambroxol, diltiazem, and amikacin, respectively. **G–I,** Molecular docking plot of STOML2 with chlortetracycline, chlorzoxazone, and dirithromycin, respectively. ALA, alanine; ARG, arginine; ASN, asparagine; ASP, aspartic acid; GLN, glutamine; GLU, glutamic acid; GLY, glycine; ILE, isoleucine; LYS, lysine; MET, methionine; SER, serine; THR, threonine; TYR, tyrosine.

The potential drugs for proteins in tiers 1 and 2 (*PP.H4* > 0.5) were evaluated. The results are as follows: (i) ASS1, with arginine (DrugBank, investigational), aspartic acid (DrugBank, approved), and citrulline (DrugBank, investigational); (ii) KRT8, with ambroxol (DSigDB, approved), diltiazem (DSigDB, approved), and amikacin (DSigDB, approved); and (iii) STOML2, with chlortetracycline (DSigDB, approved), chlorzoxazone (DSigDB, approved), and dirithromycin (DSigDB, experimental). Molecular docking was conducted for the ASS1, KRT8, and STOML2 proteins. The drugs utilized for the molecular docking process were as follows: (i) ASS1 with arginine, aspartic acid, and citrulline (Fig. 5A–C); (ii) KRT8 with ambroxol, diltiazem, and amikacin (Fig. 5D–F); and (iii) STOML2 with chlortetracycline, chlorzoxazone, and dirithromycin (Fig. 5G–I). The binding energy for each interaction was generated (Table 2). The results revealed that each drug bound to its protein targets through visible hydrogen bonds and strong electrostatic interactions. The following candidates had the lowest binding energy and highly stable binding with these three proteins: (i) arginine (−5.26 kcal/mol) with ASS1; (ii) ambroxol (−4.71 kcal/mol) with KRT8; and (iii) chlortetracycline (−7.2 kcal/mol) with STOML2.

**Table 2:** Results of protein-drug molecular docking

Protein	Drug/Compound	Binding sites	Affinity (kcal/mol)	Estimated Ki (mmol/L)	Ligand efficiency
ASS1	Arginine	3	−5.26	0.14	−0.44
	Aspartic acid	6	−4.06	1.06	−0.45
	Citrulline	7	−4.03	1.11	−0.34
KRT8	Ambroxol	2	−4.71	0.35	−0.26
	Diltiazem	4	−3.11	5.25	−0.11
	Amikacin	4	0.22	1.45	0.01
STOML2	Chlortetracycline	9	−7.2	5.28	−0.22
	Chlorzoxazone	4	−5.31	0.13	−0.48

### IHC in HPA

The HPA database provides valuable information of tissue-specific staining of proteins in patients with cancer and patients who are normal (Fig. 4K). Among the three target MR proteins, only the STOML2 protein in the tissues of patients with HCC was available on the HPA website (https://www.proteinatlas.org/ENSG00000165283-STOML2/cancer/liver+cancer#img). These images demonstrated that the STOML2 protein exhibited stronger staining and a more widespread distribution in cancer tissue compared with normal tissue, suggesting an increased expression and higher protein abundance.

## Discussion

This study identified multiple causal proteins of HCC through MR on the basis of large-scale proteomic data. Seven significant plasma proteins were found and further evaluated. Through colocalization, we identified three proteins, ASS1, KRT8, and STOML2, as potential reliable targets at a holistic level. The ASS1 protein plays a crucial role in the arginine biosynthetic pathway (31). It is an enzyme involved in the urea cycle, converting ammonia into urea in the liver of uremic animals (32). KRT8 is associated with diseases such as cryptogenic cirrhosis and familial cirrhosis (33). It plays a role in keratinization and nervous system development (34). Along with KRT19, it helps connect the contractile apparatus to dystrophin at costameres in striated muscle (35). STOML2 is a mitochondrial protein that likely regulates mitochondrial biogenesis and activity (36). It enhances cardiolipin biosynthesis, binds to cardiolipin-rich membranes, and recruits and stabilizes proteins such as prohibitin, potentially contributing to the formation of functional microdomains in mitochondrial membranes (37).

There are currently several studies investigating the associations between these three proteins and tumors. One study reported that the overexpression of ASS1 is due to the upregulation of the arginine synthesis pathway (38). Additionally, ASS1 may contribute to the metastasis of gastric cancer (39). Another study suggested that STOML2 enhances mitophagy by interacting with and stabilizing PINK1, which promotes HCC metastasis (40). Furthermore, another team proposed that STOML2 can suppress the invasion ability of cancerous cells in human liver tissue by inhibiting the NF-κB pathway (41). Our study provides novel supporting data on these claims on the basis of large-scale proteomics data. With respect to KRT8, previous studies have suggested its potential role in gastric cancer (42) or prostate cancer (43). However, only one study has reported a potential association between the KRT8/KRT18 ratio and liver cancer (44). In summary, research on these proteins in liver cancer remains limited. The mechanisms by which they operate have not been fully elucidated, with current findings primarily highlighting specific observations and indications related to these proteins. Our findings offer substantial new evidence for the potential roles these proteins may play in the formation and development of liver cancer.

In our exploration of the scRNA-seq dataset, we discovered that three proteins, ASS1, KRT8, and STOML2, were expressed primarily in HPCs and malignant cells. The original authors identified HPCs as a cell population that has the potential to transform into malignant cells (12). These three proteins may facilitate the formation of malignant hepatic cells. Notably, KRT8 was significantly expressed in HPCs but not in malignant cells. Conversely, the HCC malignant cell marker AFP was exclusively expressed in malignant cells. These findings suggest that, compared with AFP, KRT8 may play a significant role in the formation of tumorous liver tissue and could even serve as an earlier biomarker for HCC. In most cases, KRT8 functions in maintaining cell structure and cellular differentiation. Therefore, when its expression is altered, KRT8 may facilitate the proliferation of abnormal progenitor cells in the liver, which later transform into liver cancer cells. Our study suggests that KRT8 acts as a detrimental protein in HCC by facilitating the formation of malignant liver cells in the early stages. The IHC staining images on the HPA was only available for STOML2, which demonstrated that the staining was stronger and had a more widespread pattern in cancer liver tissue than normal liver tissue. STOML2 thus might have an important role in HCC. ASS1 and KRT8, on the other hand, were previously unreported by other studies. These two proteins were identified as novel plasma proteins in this study and are worthy of further experimental validation.

We discovered potential drugs for three proteins associated with liver cancer: ASS1 (arginine, aspartic acid, and citrulline), KRT8 (ambroxol, diltiazem, and amikacin), and STOML2 (chortetracycline, chlorzoxazone, and dirithromycin). Our molecular docking analysis revealed that ambroxol exhibited the strongest binding with KRT8 in terms of binding energy. Ambroxol is known not only for its original function as a secretolytic agent but also for its ability to enhance the anticancer effect of microtubule-stabilizing drugs in lung carcinoma (45). Similarly, chlortetracycline exhibited the most stable binding with STOML2 and was found to reduce the invasive properties of breast cancer cells (46). Interestingly, all three potential drugs for ASS1 are amino acids, and the ASS1 protein acts as a catalyst in the arginine biosynthetic pathway. These findings present promising avenues for the future therapeutic treatment of liver cancer.

On the basis of our study results, we suggest potential directions for future therapeutic treatments for liver cancer. KRT8 is highly expressed in precancerous cells and may serve as a more effective early detection marker than AFP. Further investigations into the mechanism of KRT8 and its potential for intervention in liver cancer are warranted on the basis of similar studies conducted in other cancers. STOML2 is a protein that plays a role in regulating mitochondrial metabolism, making it valuable to explore how to intervene in tumor cells’ energy metabolism to control tumor development (47). Additionally, ASS1, a mitochondrial metabolic enzyme, also reflects the activity of tumor cells. These potential mechanisms offer new insights for future pathophysiologic research on liver cancer.

In terms of the strengths of this study, large-scale proteomic data were utilized to identify plasma proteins as biomarkers for therapeutic targets associated with liver cancer. In addition to proteomics, transcriptomic scRNAseq data were utilized to uncover the potential roles that plasma proteins play in the mechanisms underlying the formation and progression of HCC. Molecular docking analysis provided valuable insights into the development of novel drugs. However, there are also limitations to this study. Through the analysis of the scRNAseq dataset, we observed that MR proteins exhibit transcriptomic expression in liver cancer cells, suggesting that plasma MR protein expression partially originates from liver tissue at least. However, contributions from other tissues cannot be ruled out. We used UKB-PPP proteomic data as discovery data in our MR analysis, but unfortunately, there were no corresponding external pQTL data available for validation. Additionally, the discovery data in this study focused solely on the European population, so data from other populations will be necessary for further validation. In this study, we used two HCC outcome GWAS databases for analysis because the available public data resources are insufficient. We acknowledge the limited datasets is a shortcoming of this study. More large-scale proteomic studies are needed to confirm findings in this study when more liver cancer outcome options are available in the future. Notably, this study is a multiomic data analysis project, and the acquisition of actual samples or tissues were not included. Experimental validation is required to confirm the findings of this data analysis study in the future.

### Conclusion

Our study successfully identified several novel plasma proteins causally associated with HCC via MR with plasma proteomics pQTL data and single-cell RNAseq data. Furthermore, we evaluated potential therapeutic drugs for the proteins identified through our MR analysis using molecular docking. This study provides new insights and data support for future research on the precise diagnosis and treatment of HCC.

## Supplementary Material

Supplementary Data Supplementary Data
